# Clinical Outcomes of Rituximab Infusion Among Refractory Myasthenia Gravis Patients in the Philippines: A 10-Year Retrospective Study

**DOI:** 10.7759/cureus.77888

**Published:** 2025-01-23

**Authors:** Alyssa Pauline C Co, Ludwig F Damian

**Affiliations:** 1 Neurology, St. Lukes Medical Center, Quezon City, PHL

**Keywords:** acetylcholine receptor, autoantibodies, immunosupression, low- and middle-income country (lmic), musk antibody, myasthenia gravis, neuromuscular junction, refractory myasthenia gravis, rituximab, thymectomy

## Abstract

Background: Myasthenia gravis (MG) is an autoimmune disorder causing muscle weakness, with 10%-20% of cases becoming refractory to standard treatments. Rituximab, a CD20-targeting monoclonal antibody, has shown promise in refractory MG but lacks data from low- and middle-income countries (LMICs), such as the Philippines.

Methods: A retrospective, cross-sectional study was conducted in treatment-refractory MG patients in St. Luke’s Medical Center in the Philippines between January 2014 and December 2023. Clinical outcomes were assessed before and after treatment, including MGFA scores, pyridostigmine dosage, and CD19/CD20 levels. Subgroup analyses were performed based on thymectomy and MG antibody status.

Results: Twenty-one MG patients (10 acetylcholine receptor (AChR) positive, eight seronegative, and three muscle-specific kinase (MuSK) positive) were identified. These patients (mean age 46.4 years, 76% female) showed significant improvement post-rituximab, with 50% achieving complete or near-complete remission (MGFA Class I). Pyridostigmine dosage decreased significantly, and CD19/CD20 levels dropped markedly. MuSK-positive patients responded most rapidly, and thymectomy did not considerably impact outcomes. Rituximab was well-tolerated, with mild infusion-related reactions in two patients.

Conclusion: Rituximab is a safe and effective option for refractory MG, especially in MuSK-positive patients, offering a cost-effective alternative in LMICs where treatments like eculizumab are inaccessible.

## Introduction

Myasthenia gravis (MG) is an autoimmune disorder characterized by fatigable muscle weakness due to impaired neuromuscular transmission. B-cells mediate it and involve antibodies targeting the acetylcholine receptor (AChR), muscle-specific kinase (MuSK), lipoprotein-related protein 4 (LRP4), or agrin at the postsynaptic membrane of the neuromuscular junction (NMJ). Patients are classified into subgroups based on serum antibodies and clinical features, which inform therapeutic approaches and prognosis.

In patients with generalized MG (GMG), uncontrolled complement activation leads to morphologic damage of the postsynaptic membrane, resulting in neuromuscular transmission failure. Anti-acetylcholine receptor (AChR) autoantibodies impair signaling through three primary mechanisms: first is antigenic modulation wherein autoantibodies crosslink AChRs, stimulating their internalization and degradation, second is functional blockade where autoantibody binding prevents acetylcholine from attaching to its receptor, and lastly, is chronic complement activation which is the primary cause of damage to the postsynaptic membrane. The binding of anti-AChR autoantibodies to AChRs initiates the complement cascade. The cleavage of C5 into C5a and C5b triggers the terminal complement pathway, ultimately forming the membrane attack complex (MAC). The MAC-induced damage to the muscle membrane and the resulting loss of AChRs at the NMJ lead to neuromuscular transmission disruption, contributing to the muscle weakness characteristic of GMG. Conventional treatments for GMG aim to reduce autoantibody concentrations and mitigate disease progression [1].

Conventional treatments for MG focus on immunosuppressive therapies and supportive care. Corticosteroids are typically the first-line treatment; however, their long-term use can lead to significant side effects, necessitating the use of steroid-sparing agents like azathioprine and mycophenolate mofetil (MMF). While these agents may take months to show benefits, they help reduce dependence on corticosteroids. Cyclophosphamide has been a valuable option for treating refractory MG in the past, but its use is limited by severe side effects, including myelosuppression, an increased risk of infections, and hemorrhagic cystitis. For severe cases or acute exacerbations, rescue therapies, such as intravenous immunoglobulin (IVIG) and plasma exchange, are employed to provide rapid symptom relief by removing harmful antibodies, often in combination with other treatments [2]. However, 10%-20% of patients do not achieve adequate disease control with these therapies. Treatment-refractory MG is characterized by insufficient response to optimal doses of steroids and at least one immunosuppressive agent, or by disease relapse during tapering of immunosuppressive therapy.

While most MG patients achieve disease control, the burden of illness among those with refractory disease remains poorly understood and likely underestimated. Several factors contribute to treatment resistance, including alternative antibody profiles (e.g., anti-MuSK, anti-LRP4) or AChR-negative status, which may limit responsiveness to standard therapies. Thymic abnormalities, such as thymomas, thymic hyperplasia, or incomplete thymectomy, can also play a role, as can comorbidities like infections, autoimmune diseases, or symptom-exacerbating medications (e.g., antibiotics, statins). Severe or long-standing MG, particularly with bulbar or respiratory involvement, is more resistant to treatment, and some patients fail to respond adequately to immunosuppressive therapies or develop resistance over time. Dysfunctional immune regulation, characterized by defective T-cell function or cytokine imbalances (e.g., TNF-alpha, IL-6), further sustains disease activity, while genetic or epigenetic factors may alter immune responses and neuromuscular function, compounding resistance [3-9].

Refractory MG represents a significant challenge in treatment due to its resistance to standard therapies, necessitating the use of costly advanced treatments. Among the newer biological therapies, eculizumab and efgartigimod have emerged as options for patients with anti-acetylcholine receptor antibody-positive generalized MG. Eculizumab, a monoclonal antibody targeting terminal complement activation, is approved for refractory MG in AChR-antibody positive adults; however, it can cost up to US $500,000 annually. Ravulizumab, efgartigimod, rozanolixizumab, and zilucoplan are emerging treatments for MG, each presenting unique cost implications and accessibility challenges. Efgartigimod, for instance, has been reported to cost between $93,597.84 and $187,195.65 per infusion cycle, significantly higher than traditional therapies. In contrast, rozanolixizumab and zilucoplan are still under investigation, with their costs not yet fully established but anticipated to be similarly high due to their novel mechanisms of action. The prices of these therapies pose significant barriers to access, particularly in low- and middle-income countries (LMICs), like the Philippines, where healthcare systems struggle to accommodate such expensive treatments [4]. This situation highlights the need for more affordable alternative treatment to improve access for patients suffering from this debilitating condition.

Rituximab is a chimeric monoclonal antibody that targets the CD20 protein, which is predominantly expressed on the surface of B-lymphocytes. Its mechanism of action involves the depletion of these B-cells, except for plasma cells, thereby reducing the production of pathogenic autoantibodies that contribute to the autoimmune response in MG. By binding to CD20, rituximab initiates several processes, including antibody-dependent cellular cytotoxicity and complement-mediated lysis, leading to a significant reduction in B-cell populations. This depletion helps to diminish the overall autoimmune activity against the NMJ, ultimately improving muscle strength and reducing symptoms in patients with refractory MG. Additionally, rituximab may induce regulatory T-lymphocytes, further enhancing its immunomodulatory effects. Its ability to provide a rapid onset of action compared to traditional immunosuppressants makes it a valuable option for patients who do not respond adequately to standard therapies.

Rituximab has demonstrated efficacy and safety in various CD20-mediated conditions, including refractory and severe MG. It is also widely used off-label for other neurological disorders such as multiple sclerosis, inflammatory CNS diseases, chronic inflammatory demyelinating polyradiculoneuropathy (CIDP), and Stiff-Person syndrome [5-7]. It is increasingly being recognized as a viable treatment option for MG in LMICs, offering a promising alternative for patients who are refractory to conventional therapies. A study by Shivaram et al. which was done in a single center in India with 13 refractory MG patients treated with rituximab showed significant clinical improvement and remission. This was the only study that explored its effectiveness in the setting of LMICs.

The cumulative cost of rituximab for both induction and maintenance phases is often lower than that of many traditional treatments, which is particularly crucial in resource-limited settings where healthcare budgets are constrained. In the Philippines, the cost of rituximab varies significantly by healthcare setting. In private institutions, a treatment cycle costs approximately PHP 55,000-60,000 or USD 1,000, whereas in government facilities, it is more affordable at PHP 15,000-20,000 or USD 300 per cycle. Additionally, government charity programs may provide it free of charge, depending on availability. Additionally, the convenience of rituximab's dosing schedule, which requires fewer injections than many other therapies, enhances patient compliance and overall treatment outcomes [5-11].

MG is frequently encountered in both outpatient and inpatient healthcare settings; however, comprehensive local data on its disease burden remains limited due to a lack of focused studies. A local study examined the clinical profiles of Filipino MG patients at two tertiary hospitals, correlating these profiles with the presence of acetylcholine receptor (AChR) and muscle-specific kinase (MuSK) antibodies, but found no significant association between AChR antibodies and clinical manifestations. Distinguishing between thymomatous and non-thymomatous MG is essential, as thymomatous patients often experience more severe complications. A subsequent study by De Roxas et al. indicated that both thymomatous and non-thymomatous MG patients who underwent thymectomy had higher rates of complete stable remission and pharmacologic remission compared to those who did not have surgery [12].

To date, no studies in the Philippines have evaluated the clinical outcomes of refractory MG patients, regardless of antibody status, treated with rituximab. Additionally, outcomes of thymomatous MG patients who received rituximab preoperatively before thymectomy remain unexplored. This study seeks to address these gaps, making it the first in Southeast Asia and the second in Asia to assess rituximab’s effectiveness in MG patients. By doing so, it aims to provide evidence for rituximab’s potential as a more accessible and cost-effective therapeutic alternative in LMICs, where access to advanced MG treatments remains limited.

## Materials and methods

A retrospective, cross-sectional study was carried out, involving a cohort of patients with MG from a single tertiary hospital in the Philippines. Our study included adult patients over 18 years old who are clinically diagnosed with MG, who fulfilled the criteria for refractory MG using Mantegazza criteria which defines refractory MG as (1) inadequate response to maximal doses of steroids and at least one immunosuppressive drug, (2) inability to reduce immunosuppressive therapy without relapse or ongoing need for treatments like IVIG or plasma exchange, (3) severe or intolerable side effects from immunosuppressive medications, (4) comorbid conditions that limit therapy options, and (5) frequent myasthenic crises despite treatment, and received rituximab infusions between January 2014 and December 2023; patients who were lost to follow-up for at least one month after rituximab, pediatric patients, and those with ocular MG or MGFA I subtype were excluded.

The following data were collected: age at the start of rituximab treatment, gender, disease duration, baseline and worst Myasthenia Gravis Foundation of America (MGFA) scores, MG antibody serostatus, comorbidities, history of thymectomy, occurrence of myasthenic crisis before rituximab therapy, prior crisis treatments, previous immunosuppressive treatments, baseline pyridostigmine dosage (mg/day), and CD 19 and 20 counts (if available).

To evaluate the outcomes of rituximab therapy, the MGFA score, MGFA-Post Interventional Status (MGFA-PIS), reductions in pyridostigmine and immunosuppressant doses, as well as decreases in CD 19 and 20 levels (if available), were assessed at both the initiation of rituximab and during follow-up consultations. Additionally, the presence or absence of myasthenic crisis and the outcomes of thymectomy were also documented.

The rituximab dosing regimen consisted of an initial 500 mg intravenous infusion on Day 0, followed by a clinical evaluation and measurement of CD19/20 levels (cells/cu.mm) two weeks later (if available). A CD19/20 count of 20 ± 5 cells/cu.mm was used as the threshold to determine the need for a maintenance dose every six months, based on the patient's clinical response. Baseline laboratory tests, including urinalysis, hepatitis profile, AST, ALT, BUN, creatinine, chest x-ray, complete blood count, and primary immunodeficiency panel, were performed prior to the infusion to ensure the patient had no contraindications. Informed consent was obtained from all participants. Categorical variables were presented as frequencies, while continuous variables were summarized using the mean, median, and minimum-maximum values.

## Results

A total of 898 patients were diagnosed with MG at our institution between January 2014 and December 2023. Of these, 26 patients (2.9%) met the criteria for refractory MG, and all were treated with rituximab. However, five patients were lost to follow-up, leaving 21 patients who were included in this analysis. The baseline characteristics of these patients are presented in Table 1.

**Table 1:** Baseline characteristics RTX: Rituximab, AChR (+): acetylcholine receptor antibody positive, MuSK (+): muscle-specific kinase, seronegative: absence of specific antibodies, MGFA: Myasthenia Gravis Foundation of America, MGFA Class I: Any degree of weakness affecting ocular muscles, including potential weakness in eye closure, Patients with generalized muscle weakness (classes II: mild, III: moderate, and IV: severe) can be further subclassified as A or B: Class A: Symptoms are predominantly generalized, Class B: Symptoms are predominantly bulbar. PE: Plasma Exchange, MPPT: Methylprednisolone Pulse Therapy, Pred: Prednisone, MMF: Mycophenolate Mofetil, AZA: Azathioprine

	Patient 1	Patient 2	Patient 3	Patient 4	Patient 5	Patient 6	Patient 7	Patient 8	Patient 9	Patient 10	Patient 11	Patient 12	Patient 13	Patient 14	Patient 15	Patient 16	Patient 17	Patient 18	Patient 19	Patient 20	Patient 21
Age at RTX	54	20	29	62	60	55	40	18	30	58	60	47	79	20	38	38	55	29	73	63	48
Gender	F	F	F	F	M	M	F	F	F	F	F	F	M	F	F	F	F	F	M	M	F
Antibody status	-	-	-	-	AChR	AChR	-	-	-	AChR	AChR	AChR	-	AChR	MuSK	MuSK	MuSK	AChR	AChR	AChR	AChR
Disease duration (mos)	144	108	144	108	84	72	84	108	60	72	72	72	60	132	36	36	36	96	36	36	24
No. of crisis before RTX	0	1	0	0	0	1	0	1	1	0	1	0	1	0	0	0	0	0	1	0	0
Treatment of crisis	-	PE	-	-	-	MPPT	-	MPPT	PE	-	PE	-	IVIG	-	-	-	-	-	IVIG	-	-
Thymectomy		Yes	Yes			Yes				Yes	Yes			Yes				Yes			Yes
Baseline MGFA	IIIA	IIIB	IIB	IIB	IIB	IIB	IIIA	IIB	IIIA	IIIA	IIIB	IIIA	IIB	IIA	IIA	IIA	IIA	IIA	IIA	IIA	IIA
Worst MGFA grade	IVA	IVB	IIIB	IIIB	IIIB	IIIB	IVA	IIIB	IVA	IVA	IVB	IVA	IIIB	IIIA	IIIA	IIIA	IIIA	IIIA	IIIA	IIIA	IIIA
Baseline immunosup- pressants	Pred	Pred	Pred	Pred	Pred	Pred	Pred	Pred	Pred	Pred	Pred	Pred	Pred	Pred	MMF	Pred	MMF	Pred	Pred	Pred	AZA
Baseline pyridostigmine dose (mg/day)	240	360	360	240	360	360	180	180	360	150	240	240	180	180	90	240	120	240	90	240	240

The mean age at rituximab initiation was 46.4 (SD: 17.9) years, with a female predominance (76%). Thirty-eight percent of patients had MGFA class IIA, 29% had class IIB, 24% had class IIIA and 9% were class IIIB. The majority of the patients (48%) had positive AchR antibodies. The median MG duration was 72 months before rituximab initiation. Only three patients had significant comorbidities one being diagnosed with thymic carcinoma, and two with uncontrolled type 2 diabetes mellitus. The other patients had chronic obstructive pulmonary disease (COPD), asthma, dyslipidemia, diabetes, pulmonary tuberculosis (PTB), gout, and benign prostatic hyperplasia (BPH). Before rituximab treatment, the majority of patients were previously treated with prednisone while others had methylprednisolone infusion, MMF, plasma exchange, and IVIG infusion. Nine out of 21 patients (43%) underwent thymectomy, eight of which had thymomatous MG on histopathology.

Table 2 summarizes the outcomes post-rituximab infusion. In terms of MGFA scores, there is a notable shift from higher severity classes to lower ones. Half of the patients achieved MGFA class I, indicating complete or near-complete remission of symptoms. The remaining half were classified as MGFA class IIa or IIb, reflecting only mild residual weakness. Notably, only one patient remained in class IIIa, suggesting moderate generalized weakness but still a marked improvement compared to their pre-treatment severity levels.

**Table 2:** Outcomes post rituximab infusion MGFA-PIS: Myasthenia Gravis Foundation of America (MGFA) Post-Intervention Status classifies patient status as remission (complete stable or pharmacological), minimal manifestations or symptomatic. Complete stable remission (CSR): The patient has had no symptoms or signs of MG for at least one year and has received no therapy for MG during that time. There is no weakness of any muscle on careful examination by someone skilled in the evaluation of neuromuscular disease. Isolated weakness of eyelid closure is accepted. Pharmacologic remission (PR): Same for complete stable remission except that the patient continues to take some form of therapy for MG. Patients taking cholinesterase inhibitors are excluded from this category because their use suggests the presence of weakness. Minimal manifestations (MM): The patient has no symptoms of functional limitations from MG but has some weakness on examination of some muscles. This class recognizes that some patients who otherwise meet the definition of CSR or PR do have weaknesses that is only detectable by careful examination. MM-0: The patient has received no MG treatment for at least one year. MM-1: The patient continues to receive some form of immunosuppression but no cholinesterase inhibitors or other symptomatic therapy. MM-2: The patient has received only low-dose cholinesterase inhibitors (<120mg Pyridostigmine per day) for at least one year. MM-3: The patient has received cholinesterase inhibitors or other symptomatic therapy and some form of immunosuppression during the past year.

	Patient 1	Patient 2	Patient 3	Patient 4	Patient 5	Patient 6	Patient 7	Patient 8	Patient 9	Patient 10	Patient 11	Patient 12	Patient 13	Patient 14	Patient 15	Patient 16	Patient 17	Patient 18	Patient 19	Patient 20	Patient 21
Baseline MGFA	IIIA	IIIB	IIB	IIB	IIB	IIB	IIIA	IIB	IIIA	IIIA	IIIB	IIIA	IIB	IIA	IIA	IIA	IIA	IIA	IIA	IIA	IIA
MGFA post RTX	IIA	IIB	IIB	I	I	I	IIA	I	IIA	IIA	IIB	IIIAA	IIB	I	I	I	I	IIA	I	I	I
MGFA-PIS	MM-2	MM-3	MM-2	MM-2	MM-3	MM-3	MM-2	MM-2	MM-2	MM-2	MM-2	MM-2	MM-1	MM-1	CSR	CSR	CSR	MM-2	CSR	MM-2	MM-2
Post RTX pyridostigmine dose (mg/day)	120	300	120	120	240	240	90	90	120	120	120	120	90	90	0	0	0	120	0	120	120
No. of crisis after RTX	None	None	None	None	None	None	None	None	None	None	None	None	None	None	None	None	None	None	None	None	None
Add on drug apart from steroids at last visit	No	No	No	No	No	No	No	No	No	No	No	No	No	No	No	No	No	No	No	No	No
No. of RTX infusion/Cycle	6	13	13	5	9	2	1	1	1	3	2	1	1	2	3	5	2	2	1	1	1
Time to improvement in months	6	8	12	4	7	6	1	1	1	3	2	1	1	5	6	7	6	4	1	1	1
Baseline CD 19 (Count cells/cu.mm)	241	521	387	None	None	30	556	110	None	533	155	144	None	82	62	138	246	None	49	None	936
Post RTX CD 19 (Count cells/cu.mm)	3	34	1	None	None	35	232	7	None	2	None	None	None	None	13	20	17	None	95	None	46
Baseline CD 20 (Count cells/cu.mm)	231	521	387	None	None	29	532	100	None	517	144	120	None	82	63	126	246	None	44	None	936
Post RTX CD 20 (Count cells/cu.mm)	2	34	1	None	None	35	196	6	None	0	None	None	None	5	20	8	None	None	78	None	46
Adverse reaction	None	None	None	None	None	None	None	None	None	None	None	None	None	None	Rash	Rash	None	None	None	None	None

The MGFA-PIS was also used as it reflects functional improvements and tracks treatment response over time. Based on the MGFA-PIS scale, all patients demonstrated clinical improvement following treatment, with many transitioning to lower severity classes, reflecting positive therapeutic responses. Among the cohort, four out of 21 patients (19%) achieved complete stable remission (CSR), characterized by the absence of symptoms or signs of MG for at least one year without therapy, aside from isolated eyelid closure weakness, which is accepted under the criteria. The majority of patients exhibited minimal manifestations (MM), where they experienced no functional limitations but demonstrated mild weakness in certain muscles upon examination. Specifically, two patients were classified as MM-1, requiring ongoing immunosuppression (e.g., azathioprine) but no cholinesterase inhibitors or symptomatic therapies. Over half of the patients were MM-2, maintaining stability with only low-dose cholinesterase inhibitors (<120 mg pyridostigmine/day) for at least a year. Three patients fell into the MM-3 category, indicating the use of both cholinesterase inhibitors or symptomatic therapies alongside immunosuppression in the past year. In terms of pyridostigmine dosage, there was a reduction in the mean daily dose, decreasing from 232.9 ± 87.4 mg to 117.1 ± 71.7 mg.

Out of the 21 patients, 15 underwent baseline CD19 and CD20 level assessments due to cost considerations. The mean CD19 level at baseline was 279.3 cells/cu.mm, while the mean CD20 level was 271.9 cells/cu.mm. After RTX infusion, both markers showed a reduction, with post-treatment mean levels decreasing to 42 cells/cu.mm for CD19 and 35.9 cells/cu.mm for CD20, underscoring the treatment's effectiveness in depleting B-cell populations.

In terms of duration, the average time to improvement is four months. In terms of adverse reactions, two patients, both MuSK-positive, experienced infusion-related reactions during the induction phase, presenting with rashes. These reactions were effectively managed by administering antihistamines and slowing the infusion rate. Notably, none of the patients experienced significant long-term side effects, such as cytopenia, or opportunistic infections like progressive multifocal leukoencephalopathy (PML), following rituximab infusion. This underscores the favorable safety profile of the treatment. None of the patients had a myasthenic crisis nor needed an add-on drug post-RTX infusion.

We conducted a subgroup analysis to compare the outcomes of patients who underwent thymectomy combined with rituximab versus those who received medical management alone as seen in Table 3. Demographically, thymectomy patients were younger, with a mean age of 42.4 years, and predominantly AChR antibody-positive, whereas medically managed patients had a mean age of 49.3 years and were predominantly MuSK-positive and seronegative. At baseline, thymectomy patients required higher daily pyridostigmine doses and had higher CD19/CD20 counts compared to medically managed patients. Following rituximab infusion, the time to clinical improvement was slightly longer in the thymectomy group (4.6 months) compared to the medically managed group (3.5 months). Both groups demonstrated improvement, shifting from higher severity to lower severity on the MGFA scale. In terms of MGFA-PIS, the majority of patients in both groups required <120 mg/day of pyridostigmine post-treatment. However, some thymectomy patients continued immunosuppression (e.g., azathioprine), whereas in the medically managed group, three patients achieved complete stable remission, remaining asymptomatic and medication-free for over a year. Both groups tolerated treatment well, with no major adverse events apart from two MuSK-positive patients who developed mild rashes during induction, as previously noted. Importantly, neither group experienced myasthenic crises post-rituximab infusion, highlighting the safety and effectiveness of both approaches.

**Table 3:** Comparison of those who underwent thymectomy + rituximab vs those who received medical management alone RTX: Rituximab, AChR: acetylcholine receptor, MuSK: muscle-specific kinase, MGFA-PIS: Myasthenia Gravis Foundation of America Post Interventional Status

	Thymectomy + RTX (n=9)	Purely medical (n=12)
Demographics	Mean age: 42.4 years, 7 Females, 2 Males, Predominantly AChR (+)	Mean age: 49.3 years, 9 Females, 3 Males, Predominantly MuSK and seronegative
Pyridostigmine dose	Baseline (mean): 263.3 mg/day, Post RTX (mean): 74.5 mg/day	Baseline (mean): 210 mg/day, Post RTX (mean): 85.7 mg/day
CD 19/20 Count	Baseline (mean CD19/20): 377.7/373.7 count cells/cu.mm, Post RTX (mean): 21.8/20.2 count cells/cu.mm	Baseline (mean CD19/20): 193.3/182.8 count cells/cu.mm, Post RTX (mean): 62.3/51.7 count cells/cu.mm
Time to improvement	4.6 months	3.5 months
MGFA and MGFA-PIS post RTX	MGFA: I - 2, IIA -3, IIB - 4 MGFA-PIS: CSR -1, MM2 - 6, MM3 - 2	MGFA: I - 8, IIA - 2, IIB - 1, IIIA - 1 MGFA-PIS: CSR - 3, MM1 - 1, MM2 - 7, MM3 – 1
Adverse reactions	None	2 patients (both MuSK) had infusion-related reaction (rashes)
Myasthenic crisis post RTX	None	None

Table 4 shows the comparison of MG subtypes, and it showed distinct demographic, clinical, and treatment response patterns. MuSK-positive MG was predominantly observed in younger, female patients and was characterized by severe generalized disease affecting ocular, bulbar, and respiratory muscles. Rituximab demonstrated a good to excellent therapeutic response in this group, often resulting in long-term symptom control. In contrast, AChR-positive MG, which exhibited a bimodal distribution affecting both younger and older adults (more commonly in those with late-onset MG in their 50s or 60s), presented with ocular, bulbar, or generalized weakness and affected males and females equally. Rituximab provided moderate benefits in this subtype, improving muscle strength and significantly reducing CD19/CD20 levels. Seronegative MG, predominantly seen in older females, typically presents with bulbar weakness or generalized symptoms. While rituximab led to partial responses in this group, the time to improvement was slower (mean 6.3 months) compared to other subtypes. These findings highlight the variability in disease characteristics and rituximab efficacy across MG subtypes, with MuSK-positive MG showing the most rapid and robust improvement, whereas AChR-negative MG demonstrated slower and less pronounced responses.

**Table 4:** Comparison among MG subtypes MuSK: muscle-specific kinase, AChR: acetylcholine receptor, MG: Myasthenia gravis

	MuSK (+) (n=3)	AChR (+) (n=10)	Seronegative (n=8)
Demographics	Mean age: 32.6 years (typically younger), Female predominance	Mean age: 51.6 years (variable, both young and old), Equal distribution of males and females	Mean age: 58.7 years (typically older), Female predominance
Clinical symptoms	Severe bulbar and respiratory symptoms	Ocular, bulbar, or proximal limb weakness	Bulbar weakness or generalized symptoms
Time to improvement	4.3 mos	3.4 mos	6.3 mos
Response to RTX	Good to excellent response	Good response	Variable response (Good in some, partial in others)
Key Clinical Outcomes	Significant improvement in muscle strength, Reduction in the need for other immunosuppressants and hospitalizations, Some patients experience long-term remission or significant symptom control, Relapses possible but generally less frequent and severe after rituximab	Improvement in muscle strength, fatigability, and ocular/bulbar function, Long-term benefit with continued rituximab infusions, but not all patients achieve complete remission, Relapses possible but may be less frequent and severe in responders	Improvement in symptoms, but slower or less consistent response compared to AChR-positive and MuSK-positive MG

## Discussion

Approximately 10%-20% of MG cases are treatment-refractory, leading to a substantial disease burden and economic loss. There is an emerging need to identify safe, faster, and effective treatments for these patients. The findings from this cohort of 21 patients with refractory MG treated with rituximab provide a compelling insight into the clinical efficacy and safety of this therapeutic approach [1-3]. Given the complexities of managing refractory MG, the results of this study contribute to the growing body of evidence supporting rituximab as an effective treatment for this challenging subgroup of MG patients.

The study cohort had a mean age of 46.4 years, with a female predominance (76%), consistent with the higher prevalence of MG in women. Most patients had AchR-positive MG (48%), with others having MuSK-positive or AChR-negative MG. The median disease duration before rituximab initiation was 72 months, indicating long-standing, refractory disease.

The treatment of refractory MG has evolved, reflecting advances in immunopathology and molecular medicine. Improved clinical trial designs have provided high-level evidence supporting these novel therapies, which focus on patient-specific, targeted immunological treatments such as complement and FcRn inhibitors, B-cell depletors, and CAR-T cell therapies [13-15]. These agents offer better safety profiles compared to older broad-spectrum immunosuppressants. However, questions remain about their long-term safety, effectiveness in seronegative MG, potential as first-line treatments for treatment-naïve patients, and the optimal strategies for switching between therapies [16,17]. Additionally, patients in LMICs may not benefit from these advancements due to limited access and high costs associated with these novel treatments. Our study found clinical improvement in most patients post-rituximab, with half achieving MGFA class I (complete or near-complete remission). This supports previous findings that rituximab can induce durable remissions in refractory MG. Nearly all patients showed improvement, with some achieving complete stable remission (CSR). Additionally, the need for pyridostigmine was notably reduced, from an average of 232.9 mg to 117.1 mg daily, suggesting both improved disease control and reduced reliance on symptomatic treatment, likely due to B-cell depletion. The marked reduction in CD19 and CD20 B-cell populations following rituximab treatment is another key finding of this study. This depletion aligns with rituximab's mechanism and correlates with clinical improvement, supporting the role of B-cells in refractory MG pathogenesis. The treatment was well-tolerated, with mild infusion-related reactions (rashes) in two MuSK-positive patients, managed with antihistamines. Importantly, there were no long-term adverse effects, such as cytopenias, PML, or myasthenic crises, further emphasizing the safety and efficacy of rituximab for refractory MG.

The subgroup analysis provides additional insight into the different responses to rituximab based on patient characteristics, particularly with respect to thymectomy and disease subtypes. Thymectomy, a surgical approach often used in MG treatment, was associated with a slightly longer time to improvement, but both groups showed clinical improvement, with no major differences in MGFA outcomes between thymectomy and non-thymectomy patients, reinforcing the notion that rituximab itself is a highly effective therapy for refractory MG, regardless of whether thymectomy is performed.

The differences in clinical outcomes based on MG subtype - specifically between MuSK-positive, AChR-positive, and seronegative MG - are particularly noteworthy. MuSK-positive MG, which often presents in younger, female patients with severe generalized disease, showed the most rapid and robust response to rituximab, with many patients achieving complete or near-complete remission. In contrast, seronegative MG, typically seen in older females, had a slower and less pronounced response, with some patients requiring prolonged treatment times to see significant improvement. This suggests that while rituximab is effective across all subtypes of MG, the response may be more rapid and robust in MuSK-positive patients, who may benefit most from this treatment approach.

This study has several limitations that should be acknowledged. First, the small sample size of 21 patients and the retrospective study design restrict the ability to draw definitive conclusions about long-term outcomes. Additionally, the subgroup analysis by thymectomy status and MG subtype, while valuable, is limited by the uneven distribution of patients across these groups. Future prospective, randomized controlled trials with larger and more diverse patient populations are essential to validate these findings and evaluate the long-term efficacy and safety of rituximab in refractory MG.

## Conclusions

In conclusion, rituximab appears to be a highly effective and safe treatment for refractory MG, with most patients experiencing clinical improvement and many achieving long-term remission. B-cell depletion is clearly associated with therapeutic benefit, and the safety profile is favorable, with minimal adverse effects. The findings also suggest that the response to rituximab may vary depending on the MG subtype, with MuSK-positive patients showing the most robust and rapid improvement. These results reinforce the role of rituximab as an important therapeutic option for patients with refractory MG and provide a foundation for future studies to further refine treatment strategies for this challenging disease.

To increase the generalizability and relevance of our findings, we recommend that future research include multicenter collaborations across LMICs. Such efforts would provide a more comprehensive representation of diverse healthcare systems and patient demographics, thereby enhancing the understanding of MG management in resource-limited settings. Comparative studies examining how variations in healthcare infrastructure and access to treatments influence patient outcomes in MG across LMICs are also warranted. Moreover, while this study underscores the effectiveness of rituximab in treating refractory MG, it highlights the need for a more personalized approach to treatment. Differences in treatment responses, particularly the slower improvement seen in seronegative patients, suggest that additional research is needed to identify biomarkers or clinical predictors that could help optimize patient selection and maximize the benefits of rituximab therapy.
